# Fluorescent analysis of boar sperm capacitation process in vitro

**DOI:** 10.1186/s12958-019-0554-z

**Published:** 2019-12-19

**Authors:** Lukas Ded, Pavla Dostalova, Eva Zatecka, Andrej Dorosh, Katerina Komrskova, Jana Peknicova

**Affiliations:** 1 Laboratory of Reproductive Biology, Institute of Biotechnology, Czech Academy of Sciences, BIOCEV, Vestec, Czech Republic; 2 Department of Zoology, Faculty of Science, Charles University, Prague, Czech Republic

**Keywords:** Acrosome reaction, Fluorescent microscopy, Flow cytometry, Chlortetracycline assay, Tyrosine phosphorylation, Acrosin staining, Phalloidin staining

## Abstract

**Background:**

Capacitation involves physiological changes that spermatozoa must undergo in the female reproductive tract or in vitro to obtain the ability to bind, penetrate and fertilize the egg. Up to date, several methods have been developed to characterize this complex biological process. The goal of the presented study is to mutually compare several fluorescent techniques, check their ability to detect changes in molecular processes during the capacitation progress and determine their ability to predict the percentage of acrosome reacted (AR) sperm after the exposure to solubilized *zona pellucida* (*ZP*). The capacitation process was analyzed using four fluorescent techniques: 1. chlortetracycline (CTC) staining, 2. anti-acrosin antibody (ACR.2) assay, 3. anti-phosphotyrosine (pY) antibody assay, 4. fluorescein isothiocyanate-conjugated phalloidin (FITC-phall) assay. All these methods were tested using fluorescent microscopy and flow cytometry.

**Results:**

All selected methods are capable to detect the capacitation progress of boar sperm in vitro, but there are significant differences in their outcome when using fluorescent microscopy or flow cytometry experimental arrangements and subsequent statistical analysis (KW-ANOVA). Also, the ability to predict the absolute numbers of sperm which will undergo *ZP*-induced AR differ significantly (CTC and ACR.2 gave the best predictions).

**Conclusions:**

Our study compared four largely used methods used to characterize capacitation process, highlighted their differences and showed that all are able to detect capacitation progress, CTC and ACR.2 are furthermore able to accurately predict the percentage of AR sperm after *ZP*-induced AR.

## Introduction

Capacitation is a physiological process that spermatozoa must experience in the female reproductive tract or in vitro to obtain the ability to bind, penetrate and fertilize the egg [1–3]. The capacitation is based on many molecular processes including changes in the intracellular calcium concentration [4], rearrangement of the acrosomal matrix [5], rearrangement of the sperm cytoskeleton [6–8], phosphorylation of sperm proteins [9, 10] and changes in the sperm plasma membrane [11].

Since the discovery of capacitation, several methods have been developed to characterize this complex biological process. There are four major fluorescent methods, to be mentioned, and they all target different sperm characteristics: 1. CTC method detects the redistribution of the intracellular calcium in the sperm head during capacitation [12, 13]; 2. ACR.2 recognizes the rearrangement of the acrosomal matrix by detecting changes in the accessibility of acrosin epitopes. The higher accessibility of the acrosin epitopes is a significant marker of capacitation progress [14]; 3. FITC-phall) binds to F-actin, as actin polymerization significantly increases during capacitation progress [15]; 4. Fluorescein isothiocyanate-conjugated antibodies, such as anti-phosphotyrosine (pY) antibody (anti-pY), detecting a capacitation dependent phosphorylation of various proteins [16, 17].

All accounted methods can be used in various experimental protocols, e.g. CTC in fluorimetry, ACR.2 in ELISA, anti-Y in western blot etc. Fluorescent analysis is a general method suitable for all detection procedures and generally, there are two ways to perform fluorescent analysis on a cellular level: 1. by fluorescent microscopy and 2. flow cytometry. The physiological acrosome reaction (AR) is triggered by glycolytic extracellular matrix of the egg called *zona pellucida* (*ZP*) [18].

A standardize and reliable evaluation of capacitation and the selection of a reliable detection methods is a methodological prerequisite for the quality assessment of fertilizing potential of individual sperm and sperm population exposed to physiological or environmental factors. In our study, we focused in detailed on analyzing the capacitation process of boar sperm through fluorescent detection using both fluorescent microscopy and flow cytometry. The aim of this work was to assess the ability of individual methods to detect relevant molecular changes during sperm capacitation; to compare their advantages and disadvantages in order to select a suitable method for evaluation of sperm capacitation and estimate the potential of individual methods to predict sperm ability to undergo *ZP* triggered AR and subsequently fertilize the oocyte.

## Materials and methods

### Chemicals

All chemicals were purchased from Sigma (Prague, Czech Republic) unless otherwise specified.

### Sperm preparation, capacitation in vitro and zona pellucida-induced acrosome reaction

Boar (*Sus scrofa*) ejaculates (20 ejaculates from 20 individual animals) were supplied by Insemination Station, Kout na Sumave, CR as chilled (17 °C) and diluted samples [19]. All sperm samples were examined for motility and viability (minimal parameters to include a sample into the analysis were 80% motility, 80% viability; the actual variability of both parameters was not higher than 5% among all samples included to the analysis), washed twice in tris-buffered saline (TBS, 200 x g, 10 min), centrifuged on Percoll gradient (80, 70, 55, 40% Percoll, 200 x g, 60 min) and washed in capacitation medium without bovine serum albumine (11.3 nM NaCl, 0,3 mM KCl, 1 mM CaCl_2_, 2 mM TRIS, 1.1 mM glucose, 0.5 mM pyruvate). Sperm were resuspended in capacitation medium containing BSA (1 mg/mL) to concentration 5 × 10^7^ sperm/ml. and suspension was incubated for 60, 120, 180, 240 min under paraffin oil at 37 °C, 5% CO_2_.

After 240 min of incubation, selected samples incubated for 240 min were treated by boar solubilized *ZP* (Czech University of Life Sciences, Prague, Czech Republic) for 60 min (37 °C, 5% CO_2_) [18] to induce acrosome reaction. The percentage of acrosome reacted sperm was determined by staining the acrosomes with FITC-conjugated *Pisum sativum* agglutinin (PSA).

### CTC and indirect immunofluorescence assays

The CTC was performed as described previously [13] using the following protocol. After the capacitation process (60, 120, 180, 240 min) sperm suspensions were centrifuged at 200 x g, for 5 min; the capacitation medium was removed and kept at − 20 °C. Sperm were re-suspended in phosphate-buffered saline (PBS) and mixed with equal volume (45 μl/45 μl) of CTC solution (750 mmol/l CTC in 130 mmol/l NaCl, 5 mmol/l cysteine, 20 mmol/l Tris-HCl, pH 7.8) and incubated for 30 min. Cells were then fixed in 8 μl of 12.5% paraformaldehyde in 0.5 mol/l Tris-HCl (pH 7.4). After incubation, sperm suspension was smeared onto a glass slide covered by a cover slip. To avoid evaporation and CTC fading, slides were kept in a dark wet chamber and immediately evaluated.

ACR.2 (Exbio 11–260-C100) immunofluorescent analysis was described previously [20]. After the capacitation process, sperm suspensions from all incubation times (60, 120, 180, 240 min) were centrifuged (200 x g, 5 min); the capacitation medium was removed, and kept at − 20 °C. Sperm were re-suspended in equal volume of phosphate-buffered saline (PBS), smeared onto glass slides, dried and kept at 4 °C. During fluorescent labelling preparation, sperm slides were fixed with acetone for 10 min, rinsed with PBS, treated with ACR.2 monoclonal antibody (50 μg/ml), anti-pY antibody (Sigma-Aldrich P5872; 10 μg/ml) or FITC-phall (Sigma-Aldrich P5282; 50 μg/ml) binding specifically to actin filaments, and incubated in a wet chamber for 60 min at 37 °C. After thorough washing in PBS, the ACR.2 and anti-pY smears were treated with FITC-conjugated anti-mouse IgG antibody (Sigma-Aldrich F0257; 1:500) and incubated in a wet chamber for 60 min at 37 °C. After washing in PBS and water, smears were mounted by the Vectashield mounting medium with DAPI (Vector Lab., Burlingame, CA).

**Fig. 1:**
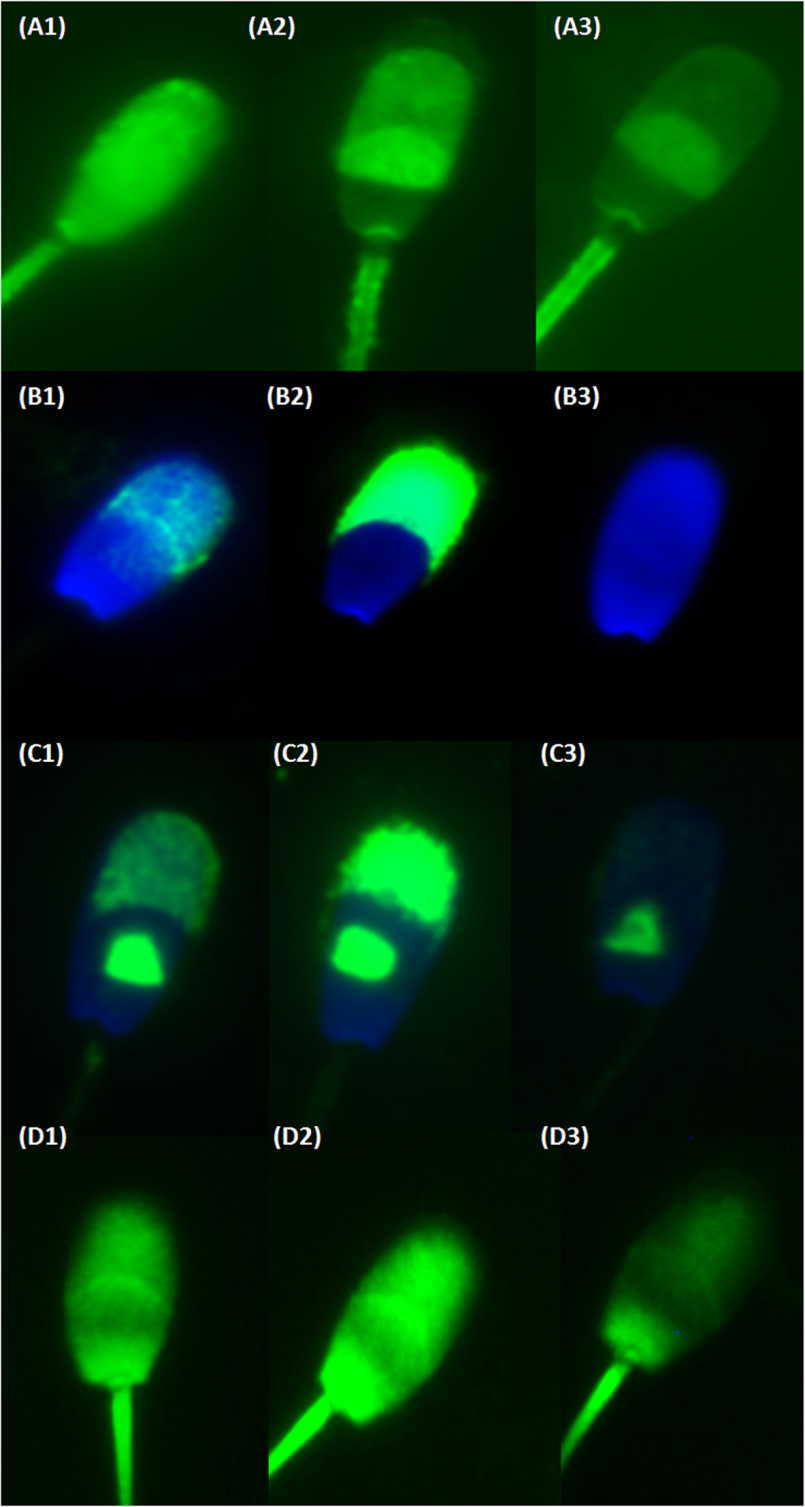
Fluorescent microscopy pictures of sperm stained with CTC, ACR.2, anti-pY and FITC-phall. Acrosomal and sperm head fluorescent patterns prominent in distinct stages of capacitation process. a1 – a3 sperm treated by CTC: a1 Non-capacitated, acrosome-intact sperm - bright fluorescence over the entire sperm head and positive mid-piece of the tail; a2 Capacitated, acrosome-intact sperm - prominent fluorescent positive equatorial segment and mid-piece, fluorescence-free (dark) band in the post-acrosomal region; a3 Acrosome-reacted sperm - low fluorescent signal throughout the sperm head, with a remaining positive signal in the equatorial segment and mid-piece. B1 – B3 representative pictures of three specific ACR.2 acrosomal fluorescent patterns: b1 Non-capacitated, acrosome-intact sperm - moderate uniform fluorescence in the acrosomal area; b2 Capacitated, acrosome-intact sperm - intensive fluorescence of the acrosome; b3 Acrosome-reacted sperm - low or no fluorescent signal in the sperm head. Anti-pY: C1 – C3 pictures of three specific pY staining patterns: c1 Non-capacitated sperm – moderate signal in the acrosomal area, visible triangular segment; c2 Intensive fluorescence of the sperm head, triangular segment and tail – capacitated, acrosome-intact sperm; c3 Very low/no signal in the acrosomal area, visible triangular segment – acrosome reacted sperm. D1 – D3 representative pictures of three specific FITC-phall staining: d1 Non-capacitated sperm – moderate fluorescence in the acrosomal and sperm head/tail area; d2 Intensive fluorescence of the acrosome and the tail – capacitated, acrosome-intact sperm; d3 Low intensity in the acrosomal and apical sperm head area – sperm after AR. b1 – b3, c1 – c3 nuclei stained with a Blue DAPI dye

Samples were examined with a Nikon Labothot-2 fluorescent microscope equipped with 40x Nikon Plan 40/0.65 and photographed with a COHU 4910 CCD camera (Inc. Electronics Division, San Diego, USA) using LUCIA imaging software (Laboratory Imaging Ltd., Prague, Czech Republic). Sperm cells were classified according to their cellular (acrosomal) staining patterns into non-capacitated, acrosome intact sperm; capacitated, acrosome-intact sperm; and acrosome-reacted sperm (Table 1; Fig. 1). In each sample, 200 cells were evaluated.

**Table 1:** Specific fluorescent patterns of the boar sperm (chilled 17 °C/diluted) as detected by individual fluorescent methods

Assay	chlortetracycline (CTC)	anti-acrosin antibody (ACR.2)	anti-phosphotyrosine (pY)	fluorescein isothiocyanate-conjugated phalloidin (FITC-phall)
Non-capacitated, acrosome intact sperm	Bright fluorescence over the entire sperm head and positive mid-piece of the tail	Moderate fluorescence in the acrosome	Low fluorescence in the sperm head, triangular segment within the head and tail	Moderate fluorescence in the sperm head and tail
Capacitated, acrosome-intact sperm	Prominent fluorescence in the equatorial segment, mid-piece of the tail and no fluorescence (dark) in the post-acrosomal region of the head	Intensive fluorescence in the acrosome	Intensive fluorescence in the sperm head, triangular segment within the head and tail	Intensive fluorescence in the acrosome and tail
Acrosome-reacted sperm	Low fluorescence over the sperm head, with remaining positive signal in the equatorial segment and mid-piece of the tail	Low or no fluorescence in the sperm head with a remaining fluorescence in the equatorial segment of the sperm head	No fluorescence in the acrosome with a remaining positive fluorescence in the triangular segment within the head and tail	Low fluorescence in the sperm head with a remaining positive signal in the tail

### Flow cytometry analysis

Sperm samples were collected at different time during capacitation process (0, 60, 120, 180, 240), then centrifuged and washed in PBS (200 x g, 5 min) and fixed by 96% ethanol at 4 °C for 30 min. After ethanol fixation, sperm were re-fixed in ethanol-acetone mixture at 4 °C (10:1) for 30 min. CTC treatment was carried out as described previously. Sperm intended for other analysis were washed three times in PBS and incubated with anti-acrosin ACR.2 antibody (50 μg/ml), anti-pY antibody (Sigma-Aldrich P5872; 5 μg/ml) and FITC-phall (Sigma-Aldrich P5282; 10 μg/ml) at 37 °C for 60 min. After the incubation with the primary antibody (ACR.2, anti-pY), sperm were washed three times in PBS and incubated with a FITC-conjugated anti-mouse IgG antibody (Sigma-Aldrich F0257; 1:1000)) for 60 min. FITC-phall samples were only kept in incubation chamber. After the incubation, all sperm samples were intensively washed in PBS (five times for 5 min) and subsequently 100 μl of the suspension was placed on 96-well plate. Flow cytometry data acquisition was performed on BD LSR II instrument (BD, Becton Drive Franklin Lakes, NJ, USA), excitation laser 488 nm, emission filters 530/40, measurement of fluorescent intensity in FITC channel. Analysis was performed using FlowJo 7.5.4. software (TreeStar Inc., Ashland, OR, USA; Additional file 2: Figure S2). The differences among individual samples in the % of cells in appropriate gates (NC – non-capacitated, C - capacitated, AR – after acrosome reaction) and arithmetic mean of the fluorescent intensity in the FITC channel (CTC) were assessed.

### Statistical analysis

Experimental data were analyzed using STATISTICA 7.0. (StatSoft CR, Prague, Czech Republic) and GraphPad 5.03. The statistical differences in the number of sperm cells with specific acrosomal status among control and experimental samples were assessed by the Kruskal–Wallis one-way analysis of variance (KW-ANOVA). Post hoc analysis was performed by the Newman-Keuls test and multiple comparisons of mean ranks. The Bland-Altman method was used to calculate the bias and its variance between the number of capacitated cells detected by individual methods after 240 min of incubation and the number of acrosome-reacted sperm after *ZP*-induced AR. The *p* value equal or lower to 0.05 was considered to be significant.

## Results

### Fluorescent microscopy detection of capacitation progress by individual methods

**Fig. 2:**
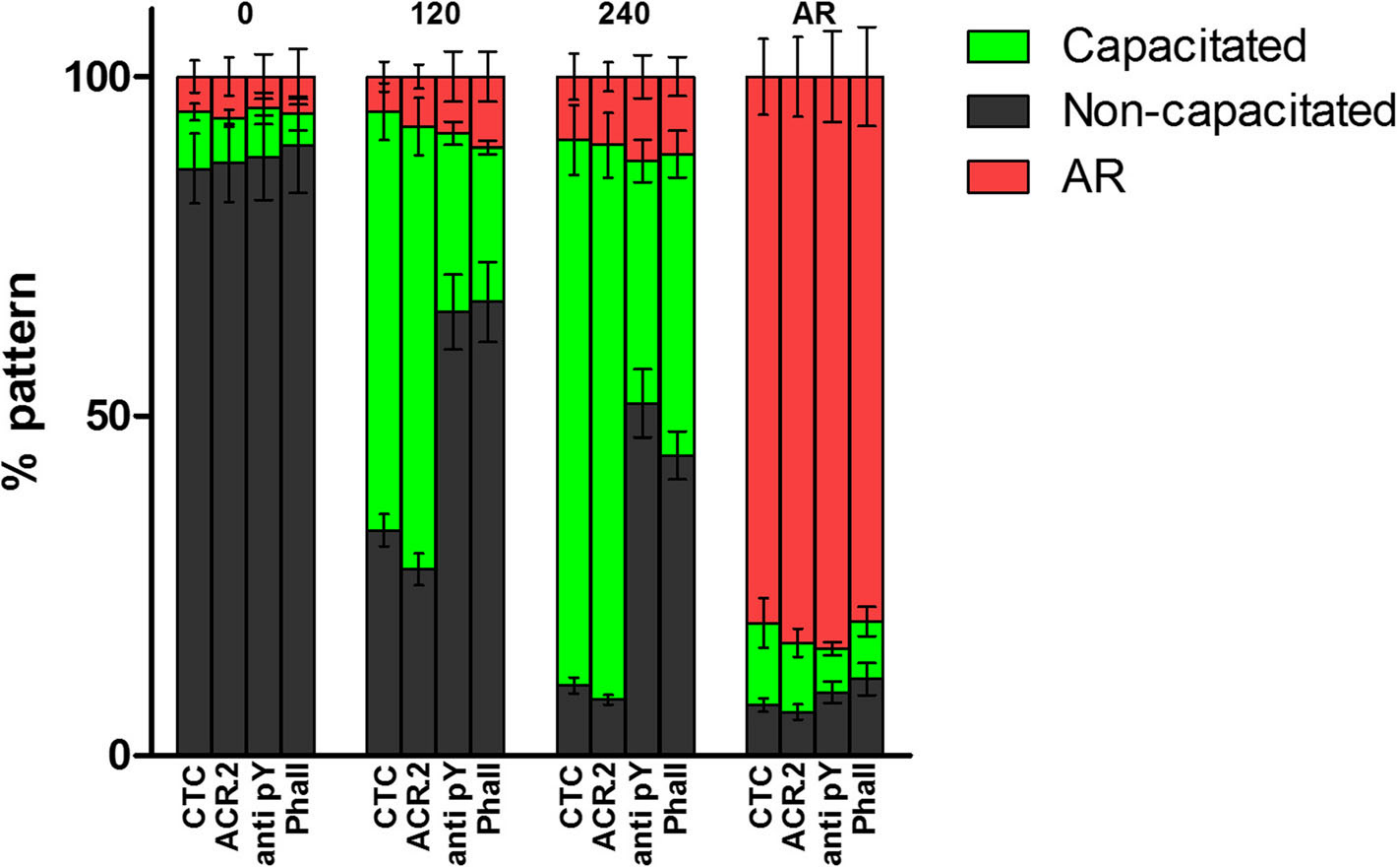
Percentage of non-capacitated, capacitated and acrosome-reacted sperm analyzed by FC after different incubation time in capacitation medium (0, 120 and 240 min) and *ZP*-induced AR. Individual bars denote the percentage of non-capacitated, capacitated and AR cells as detected by individual methods among pre-defined sequential times of the capacitation and after AR. Samples from 20 individual boars were analyzed in this assay. No significant difference among individual methods at 0 min and after AR. Significant difference between CTC/ACR.2 vs anti-pY/FITC-phall at 120 and 240 min (*p* ≤ 0.05). The percentage of capacitated sperm differed (*p* ≤ 0.05) among end points (0, 120, 240, and AR) for the same evaluation method. Error bars indicate SEM

Figures 1 and 2 summarize data from fluorescent microscopy analysis of capacitation progress by presenting the percentage of cells with specific fluorescent pattern (% pattern) as detected by CTC, ACR.2, anti-pY (also Additional file 1: Figure S1) and FITC-phall (Fig. 1) at different incubation time (Fig. 2) from 20 individual samples (*n* = 20). At the beginning of the capacitation process (time 0 min), there was 5–8% of sperm with specific fluorescent pattern evaluated as capacitated and 7% of sperm evaluated as acrosome reacted and there were no significant differences between individual methods. At 120 min, a significant increase in the number of sperm with capacitated fluorescent pattern was observed in all the methods with the highest increase in ACR.2 and CTC. Moreover, at 240 min, all the methods detected significant increase in the number of capacitated sperm. After *ZP*-induced AR, all the methods detected strong significant decrease in the number of capacitated sperm, which correlated with the sperm evaluated for the specific fluorescent staining pattern after AR.

### Flow cytometry detection of capacitation progress by individual methods

**Fig. 3:**
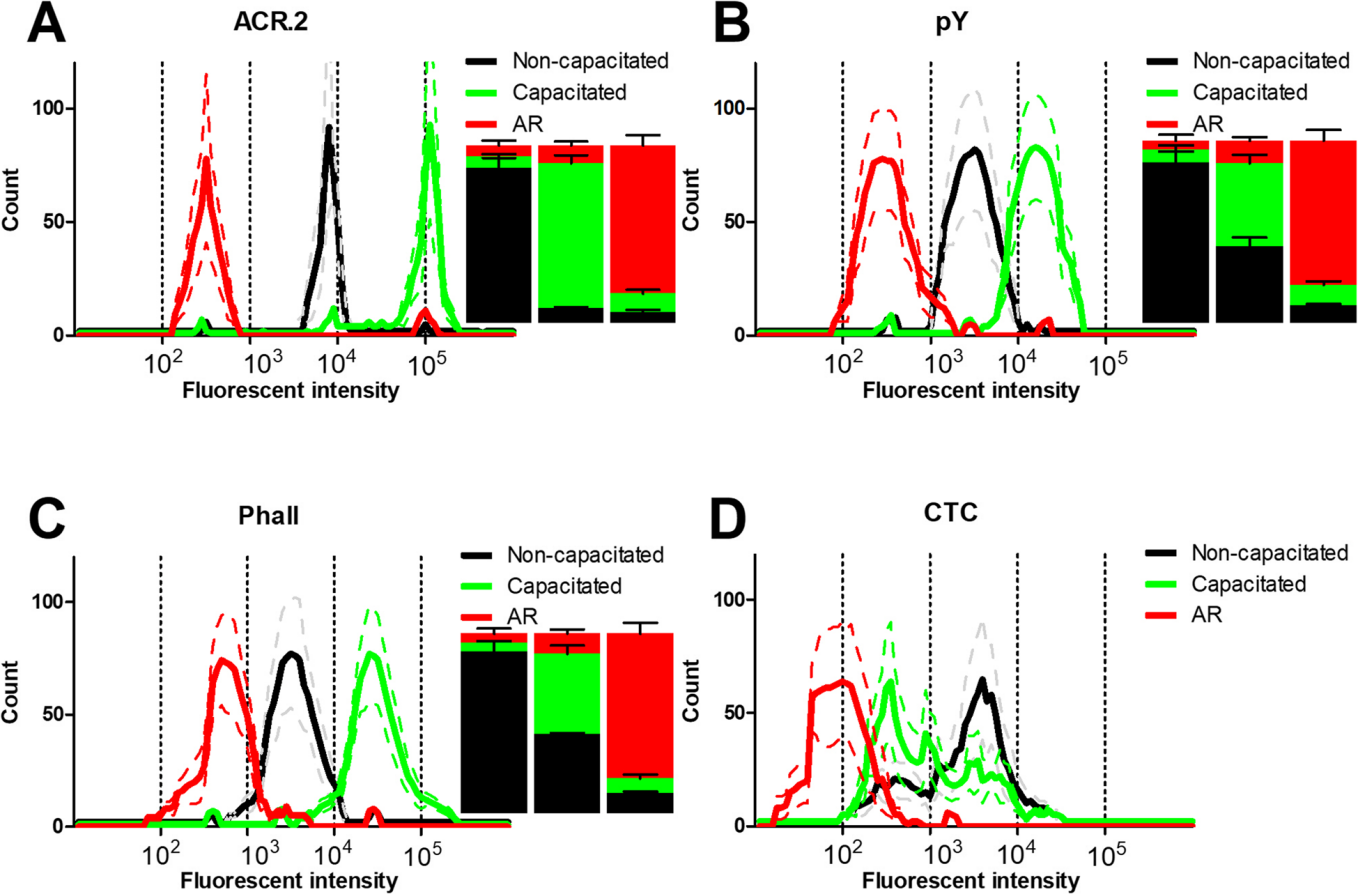
Flow cytometry histograms and percentage of non-capacitated, capacitated and AR cells. Histograms and bar charts from the flow cytometry analysis of the non-capacitated (black), capacitated (240 min; green) and AR sperm (red) as analyzed by ACR.2 (**a**), anti-pY (**b**), FITC-phall (**c**) and CTC (**d**). The histograms represent the fluorescent signal intensities in 10.000 cells in non-capacitated, capacitated and AR among the analyzed samples (*N* = 20). Inserted bars denote the corresponding distribution of the percentage of the non-capacitated, capacitated and AR cells at time 0 (left), 240 min (middle) and after AR (right). Bar graphs are not show for the CTC assay

Figure 3 summarizes data from flow cytometry analysis of capacitation progress by presenting the flow cytometry histograms of the intensity in the FITC channel and the percentages of cells in appropriate gate at the beginning of capacitation (time 0 min), at the end of capacitation (time 240 min) and after the *ZP*-induced AR. Fluorescent intensity increased in all methods during capacitation progress with the exception of the CTC assay, where fluorescent intensity was highly stochastic and was not subjected to subsequent gating and analysis (panel D was subsequently used for correlation analysis for CTC absolute fluorescent intensity). The ACR.2 detection method (Fig. 3a) displayed three completely separated peaks corresponding to the non-capacitated (NC), capacitated (C) and acrosome-reacted (AR) sperm populations and indicated the highest differences between the numbers of NC, C and AR sperm. On the other hand, pY detection method (Fig. 3b) provided three well distinguishable, but not completely separated peaks for NC, C and AR sperm populations. The overlap among individual intensity peaks also led to the smaller differences in the percentage of the individual sperm populations. The similar output was provided by the Phall detection method (Fig. 3c) with slightly higher overlap between individual intensity peaks especially for the NC/AR sperm populations.

### Correlation of individual methods

Table 2 represents the correlation between a number of capacitated sperm cells among individual detection methods. All the methods expressed significant correlation (*p* ≤ 0.05) with exception of flow cytometry for CTC. The highest correlation was observed between fluorescent microscopy detection of the capacitation state by CTC and ACR.2 antibody. Even though there was a high Pearson correlation coefficient (*r* = 0.81) for fluorescent microscopy data, the individual methods expressed different sum of correlation coefficients (r total) with the highest r total for CTC and ACR.2 fluorescent methods. In contrast, flow cytometry with CTC and fluorescent microscopy with FITC-phall had the lowest r total (*r* total = 2.51 and 3.34). In general, individual data sets from fluorescent microscopy expressed higher in-between correlation compared to coefficients between fluorescent microscopy (FM) and flow cytometry (FC) data and oppositely.

**Table 2:** Correlation matrix of individual detection methods of boar sperm (chilled 17 °C/diluted) capacitation status at 240 min of incubation; *n* = 20

	ACR.2 FM	ACR.2 FC	CTC FM	CTC FC	Phall FM	Phall FC	pY FM	pY FC
ACR.2 FM^a^	**1**	**0.79**	**0.81**	0.19	**0.33**	**0.55**	**0.47**	**0.55**
ACR.2 FC^b^	**0.79**	**1**	**0.69**	0.17	**0.33**	**0.62**	**0.39**	**0.44**
CTC FM^c^	**0.81**	**0.69**	**1**	0.25	**0.35**	**0.59**	**0.52**	**0.47**
CTC FC^d^	0.19	0.17	0.25	**1**	0.24	0.31	0.12	0.23
Phall FM^e^	**0.33**	**0.33**	**0.35**	0.24	**1**	**0.47**	0.27	**0.35**
Phall FC^f^	**0.55**	**0.62**	**0.59**	0.31	**0.47**	**1**	0.23	0.29
anti-pY FM^g^	**0.47**	**0.39**	**0.52**	0.12	0.27	0.23	**1**	**0.71**
anti-pY FC^h^	**0.55**	**0.44**	**0.47**	0.23	**0.35**	0.29	**0.71**	**1**
r total^i^	**4.69**	**4.43**	**4.68**	**2.51**	**3.34**	**4.06**	**3.71**	**4.04**

Correlation coefficients (r) between detections of capacitation status by individual detection methods

^a^Fluorescent microscopy with anti-acrosin (ACR.2 FM) antibody

^b^ Flow cytometry with ACR.2 antibody (ACR.2 FC)

^c^Fluorescent microscopy with chlortetracycline (CTC FM)

^d^Flow cytometry with chlortetracycline (CTC FC)

^e^Fluorescent microscopy with fluorescein isothiocyanate-conjugated phalloidin (Phall FM)

^f^Flow cytometry with fluorescein isothiocyanate-conjugated phalloidin (Phall FC)

^g^Fluorescent microscopy with anti-phosphotyrosine antibody (anti-pY FM)

^h^Flow cytometry with anti-phosphotyrosine antibody (anti-pY FC)

^i^The sum of correlation coefficients for appropriate detection method

Significant correlation coefficient in bold (*p* ≤ 0.05)

### Correlation between number of capacitated cells detected by individual methods and number of cells after *ZP*-induced acrosome reaction (AR)

Table 3 represents the correlation between number of capacitated cells after 240 min of incubation and number of cells after *ZP*-induced acrosome reaction. The Pearson correlation coefficients (r) between number of cells with specific acrosomal pattern and means of fluorescent intensity (detected by CTC, ACR.2, anti-pY and FITC-phall) and numbers of cells after AR (detected by PSA fluorescent microscopy (PSA FM) and flow cytometry (PSA FC)) are presented. The strongest correlation was observed between a number of capacitated cells detected by CTC fluorescent microscopy (CTC FM) and a number of cells after AR detected by fluorescent microscopy with PSA (PSA FM). Almost the same result was obtained by fluorescent microscopy with ACR.2 antibody (ACR.2 FM) and PSA FM. All other methods and approaches expressed correlation at different levels of significance with exception of flow cytometry with CTC. In general, individual data sets from fluorescent microscopy expressed higher in-between correlation compared to coefficients between FM and FC data.

**Table 3:** Correlation matrix between % of capacitated boar sperm (chilled 17 °C/diluted) after 240 min of incubation detected by individual methods and number of cells after *ZP*-induced acrosome reaction detected by PSA; *n* = 20

	PSA FM^i^	PSA FC^j^
ACR.2 FM^a^	**0.92**	**0.69**
ACR.2 FC^b^	**0.77**	**0.84**
CTC FM^c^	**0.93**	**0.72**
CTC FC^d^	0.19	0.21
Phall FM^e^	**0.68**	**0.35**
Phall FC^f^	**0.54**	**0.62**
anti-pY FM^g^	**0.5**	**0.60**
anti-pY FC^h^	**0.64**	**0.71**

Correlation coefficients (r) between detections of capacitation status by individual detection methods and detection of cells after *ZP*-induced acrosome reaction detected by appropriate methods

^a^Fluorescent microscopy (FM) with anti-acrosin (ACR.2) antibody

^b^Flow cytometry (FC) with ACR.2 antibody

^c^Fluorescent microscopy with chlortetracycline (CTC FM)

^d^Flow cytometry with chlortetracycline (CTC FC)

^e^Fluorescent microscopy with fluorescein isothiocyanate-conjugated phalloidin (Phall FM)

^f^Flow cytometry with fluorescein isothiocyanate-conjugated phalloidin (Phall FC)

^g^ Fluorescent microscopy with anti-phosphotyrosine antibody (anti-pY FM)

^h^Flow cytometry with anti-phosphotyrosine antibody (anti-pY FC)

^i^ Fluorescent microscopy with *Pisum sativum* (PSA FM)

^j^Flow cytometry with *Pisum sativum* (PSA FC)

Significant correlation coefficient in bold (*p* ≤ 0.05)

**Fig. 4:**
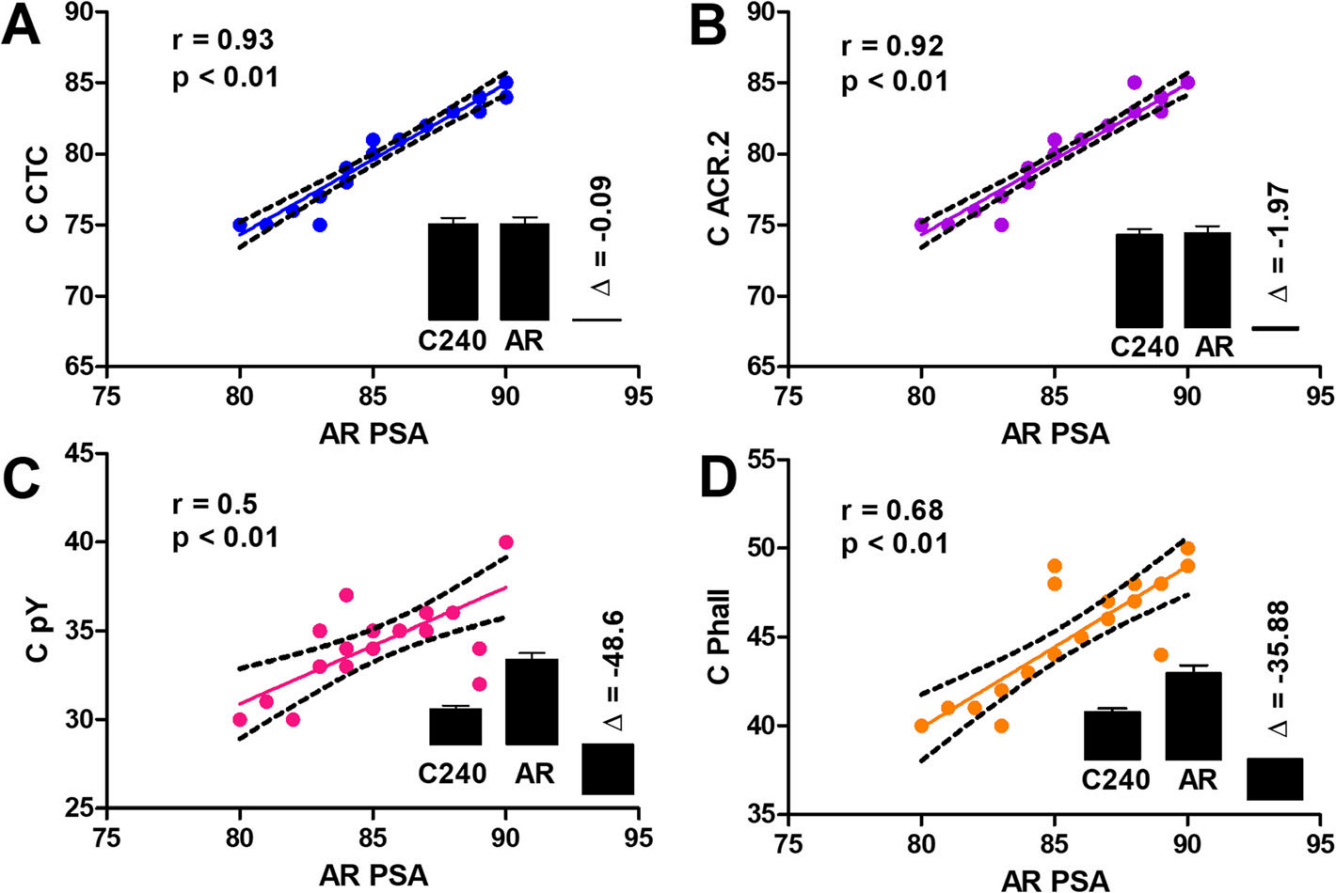
Correlations and differences between the % of capacitated boar sperm at 240 min detected by FM by individual methods and % of AR cells detected by PSA FM. Individual graphs show the correlation lines, the correlation coefficient r and its *p*-value for the CTC (**a**), ACR.2 (**b**), pY (**c**) and FITC-phall (**d**). The inserted bars represent the comparison of the percentage of cells detected as capacitated by individual methods (C240), the percentage of the cells detected as AR by PSA assay (AR) and their difference (Δ). 20 chilled (17 °C) / diluted boar sperm samples were analyzed for each assay (*N* = 20)

Figure 4 graphically summarizes the correlations between the percentages of capacitated sperm at 240 min of incubation detected by individual fluorescent microscopy methods and percentage of AR sperm after the *ZP*-induced AR detected by PSA method. The highest Pearson correlation coefficient was observed by CTC analysis (*r* = 0.93) and ACR.2 method (*r* = 0.92) while FITC-phall assay and pY assay presented moderate positive correlations (*r* = 0.68 and *r* = 0.5, respectively). All the correlation coefficients were statistically significant (*p* ≤ 0.01). Inserted bar charts (Fig. 4) represent the relative numbers of capacitated cells at 240 min of incubation detected by corresponding method (C240), the relative number of AR sperm detected by PSA assay after the *ZP*-induced AR (AR) and their differences (Δ).

**Fig. 5:**
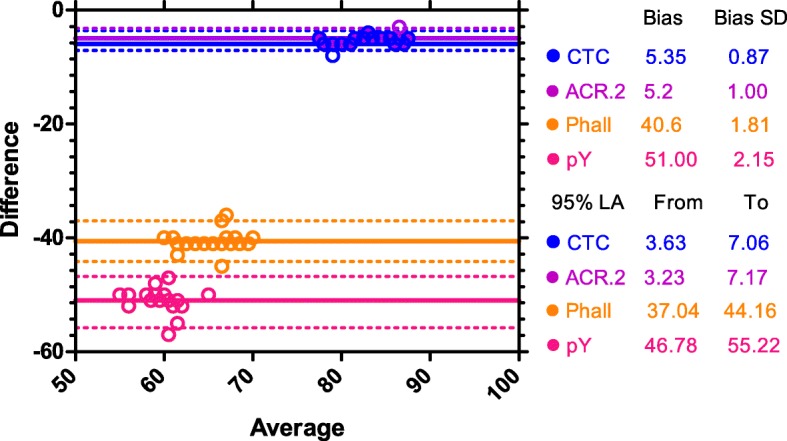
Bland-Altman plot. Bland-Altman plot shows the differential bias between the percentage of cells detected as capacitated by individual methods after 240 min of incubation and the percentage of cells detected as acrosome-reacted by PSA after *ZP*-induced AR. The zero baseline represents the percentage of cells detected as acrosome-reacted by PSA, individual color lines shows the biases for CTC, ACR.2, FITC-phall and pY assays. Circles represents individual data points (*N* = 20 for each method), dotted lines represent 95% LA (Limits of agreement)

Data from FM experiments are finally represented as a Bland-Altman plot (Fig. 5; Additional file 3: Figure S3) which shows the agreement among individual methods. The zero baseline represents the percentage of cells detected as AR by PSA. All the methods underestimated the number of acrosome-reacted sperm and there were major differences in the calculated bias for individual methods. The lowest bias between the number of cells detected as capacitated after 240 min of incubation and number of acrosome-reacted cells after *ZP*-induced AR was calculated for ACR.2 and CTC method (5.2 ± 1 and 5.35 ± 0.87), the highest bias was calculated for pY method (46.78 ± 2.15).

## Discussion

Spermatozoa have to undergo series of controlled molecular changes in female reproductive tract or in vitro before being able to bind, penetrate and fertilize the egg [1–3]. Nevertheless, many molecular and physiological aspects of capacitation are still waiting to be discovered or characterized. In our study, we targeted depiction of capacitation process dynamics by multiple fluorescent techniques and compare their detection outcome. Moreover, we were able to address the ability of individual methods to detect the measurable physiological status of capacitated sperm.

CTC is considered as gold standard in fluorescent microscopy analysis of sperm capacitation state [12, 13, 21–23]. Notable disadvantage of this method is the difficult assessment of individual cell fluorescent patterns under the fluorescent microscope [12] and a relative low fluorescent intensity in combination with fast photobleaching, which make analysis difficult for human eye. On the other hand, ACR.2 antibody analysis of the specimen is much easier for the human evaluator, due to a strong positive signal and prominent acrosomal patterns. Although anti-pY and FITC-phall are able to detect changes during capacitation in fluorescent intensity in the sperm head and tail, the major disadvantage of these methods is the absence of a specific fluorescent pattern corresponding to the capacitation progress and subsequent necessity to set an intensity threshold, which is subjective. However, this disadvantage may be overcome by using a computer picture analyzer [24]. The described challenges using anti-pY and FITC-phall methods for detection of the capacitation state resulted in lowest correlation of the data compares to those obtained by CTC and ACR.2.

The other obvious way how to overcome subjective analysis of the fluorescent intensities is to use flow cytometry. On very positive terms, in general, the flow cytometry data corresponded with those from fluorescent microscopy, with few important remarks. CTC assay may not be suitable for fluorescence detection by flow cytometry. During capacitation, the prominent change in the CTC fluorescent analysis is the appearance of the dark postacrosomal segment, a fluorescent pattern, which is not well distinguishable by cytometer detector. On the other hand, data from anti-pY and FITC-phall express much better statistical differences among individual capacitation times using flow cytometry, which might be likely due to the fact, that strong point of the flow cytometry analysis is the ability to precisely measure small differences in the fluorescent intensity. Finally, the strength of analysis using ACR.2 antibody is in the regular presence of three easily distinguishable fluorescent intensity peaks, which enables to gate them to acquire another set of useful data for statistical analysis. In general, flow cytometry generates different type of statistical parameters (e.g. arithmetical, geometrical mean of fluorescent intensity, number of accidents in set gates etc.), which are accessible for subsequent statistical analysis (e.g., comparing multiple groups by ANOVA) [17, 23, 25–27]. In our study, we analyzed the percentages of sperm in the appropriate gate for ACR.2, pY and Phall and arithmetical means of fluorescent intensity for CTC. In general, the strong point of flow cytometry analysis is ability to analyze thousands of cells per sample, objective analysis and capability to precisely measure the fluorescent intensity, which changes correlate with the physiological process. The relative weaknesses of the method are the cost of the instrument and analysis and inability to exactly asses the specific morphological fluorescent patterns, a drawback, which can be now almost overcome by sophisticated cytometers, which combine the advantages of both flow cytometers and fluorescent microscopes [28, 29].

The combination of the data from fluorescent microscopy and flow cytometry enables us to describe the temporal changes and succession of the molecular processes detected by individual analytical methods. According to our results, the first observable change is the redistribution of calcium ions (CTC FM [30];), accompanied by highest accessibility of acrosin epitopes (ACR.2 FM, FC), which resulted from enzymatic and proteomic changes in acrosomal matrix. At later capacitation stages (180 min), the phosphorylation of sperm proteins [31] and actin polymerization [6, 7] are also well detectable by presented methods. At this point it is important to mention, that collecting samples at only five different times during capacitation is not sufficient for detailed characterization of the molecular changes, on which physiological process of capacitation is based and sperm life imaging is more appropriate method to study this in detail. For example, fast changes in calcium concentration should be measured by methods other than CTC [32, 33]. Similarly, changes in actin polymerization should be measured by multiple analytical methods, since staining with FITC-phall can reflect rather changes in accessibility of actin epitopes than actin polymerization and depolymerization itself. On the other hand, CTC, contrary to methods measuring fast changes in calcium concentration, is able to reflect global changes of sperm cellular calcium homeostasis thus, likewise other methods used in this work, play important role in studying capacitation as cellular physiological process.

Due to the fact that capacitation is the physiological process, which results in the ability of sperm to undergo AR in the presence of *zona pellucida*, we tested the ability of individual methods to predict number of physiologically capacitated sperm. According to the results presented in Fig. 4 and Table 2, all used methods with well-thought of experimental design (fluorescent microscopy and flow cytometry) show a good correlation with the number of cells after *zona pellucida* induced AR, but there are major differences in their ability to predict the percentage of cells undergoing acrosome reaction in the presence of *zona pellucida* in boars. FM CTC and FM ACR.2 are best in prediction of status physiologically capacitated sperm showing the lowest bias in Bland-Altman analysis and thus can be used as a useful tool for optimization of capacitating media [34] or for studying effect of various compounds with the pro-, or anti-capacitation effect [14]. On the other hand, pY method showed the lowest agreement (the highest bias) between the number of cells detected as capacitated at 240 min and the number of cells detected as AR after *ZP*-induced acrosomal reaction and therefore in our arrangement highly underestimate the % of cells which will undergo the *ZP*-induced AR.

Despite the fact, that our experimental approach enabled to compare four methods used for characterization of capacitation process in boar sperm, and broaden up the knowledge on interpretation of obtained data, there are still several limitations, which need to be addressed in future studies. The first is related to the evaluation of individual cells in a sample by multiple analytical methods. The co-staining of the individual samples by for example ACR.2 and anti-pY antibody would enable to conclude if individual cells are detected by both methods as non-capacitated, capacitated or AR and rigorously calculate methods agreement on the level of individual cells. This approach would not be technically possible for the CTC method since sample processing and evaluation by FM differs from antibody or FITC-phall staining. The second limitation is similar but related to AR prediction. The experimental approach used in the current study also does not allow to determine if individual cells detected as capacitated by individual methods would be exactly those undergoing AR when exposed to solubilized *ZP*. The presented ratios and agreements of cells detected as capacitated by CTC and ACR.2 and cells detected as AR by PSA after *ZP*-induced AR suggest that cells detected as capacitated by these two methods will undergo AR after exposure to solubilized *ZP*. However, such conclusion can not be drawn for anti-pY and FITC-Phall methods. A possible approach to probe this in further detail would be to induced AR by *ZP* during several times of incubation where the ratios of cells detected as non-capacitated and capacitated are different and using FC observed what population of cells (non-capacitated/capacitated) will undergo AR. However, there are again several technical limitations, since ACR.2 antibody displays intermediate fluorescent intensity peaks in earlier stages of the incubation and there are gates overlaps for anti-pY and FITC-phall, as shown in Fig. 3. Similarly, the presented approach would not suit the CTC method.

To summarize, the multiple fluorescent methods used in our study to monitor boar sperm capacitation proved to be able to detect the temporal changes of the capacitation process. However, for some methods, flow cytometry is more appropriate than fluorescent microscopy and vice versa, and this should be considered in an experimental design. Data from individual analytical methods significantly correlates, albeit there are notable differences in the correlation coefficient between them. Furthermore, a change in the temporal dynamics in individual molecular processes detected by appropriate methods were observed. These individual observations and assessments are crucial, as the differences in temporal changes allow us to make rough model of chronological succession of processes underlying capacitation. Finally, using a correlation analysis with data from *ZP*-induced acrosome reaction was shown, that described methods are able to predict the number of spermatozoa undergoing AR after exposure to *ZP* but there were major differences among individual methods. The detailed knowledge of limits of these methods commonly used for evaluation of capacitation status and prediction of sperm ability to undergo the AR should help to standardize individual results and lead to production of good comparable data among scientific laboratories.

## Conclusions

Capacitation is one of the most crucial steps sperm has to undergo before being able to fertilize egg. Therefore, the proper characterization of its dynamics has great importance for many studies addressing sperm physiology. In this article, we have studied capacitation of boar sperm using four largely used methods, compared their experimental outputs using fluorescent microscopy and flow cytometry and highlighted their limits and differences when detecting capacitation progress. Furthermore, we show that CTC and ACR.2 methods are able to accurately predict the percentage of acrosome-reacted sperm after *ZP*-induced AR. Our study thus contributes further to better characterization of the important step in the mammalian reproduction such as capacitation.

## Supplementary information

**Additional file 1:** **Figure S1.** Example of the fluorescent microscopy analysis of the sperm capacitation status. The microphotograph shows 14 boar sperm stained by anti-pY antibody (green) and counterstained by DAPI. Eleven cells detected as capacitated (C), two cells detected as non-capacitated (NC), one cell as AR. Time 240 min, 400x.

**Additional file 2:** **Figure S2.** Example of the flow cytometry analysis of the boar sperm capacitation progress by ACR.2 antibody. The dot plots represent the gating of the analyzed sperm population using FSC and SSC detection and the histograms of the FITC channel with the gating of the non-capacitated (N), capacitated (C) and AR cells (%) for the individual time points (0, 30, 60, 90, 120, 180, 240) and AR. The data from 0, 240 and AR from the 20 individual boars were subsequently used for the preparation of Fig. 3.

**Additional file 3:** **Figure S3.** Bland-Altman plots separately for individual methods. Bland-Altman plots (decomposed Fig. 5) show the absolute bias between the percentage of cells detected as capacitated by individual methods after 240 min of incubation and the percentage of cells detected as acrosome-reacted by PSA after *ZP*-induced AR. The zero baseline represents the percentage of cells detected as acrosome-reacted by PSA, solid lines shows the absolute biases for CTC, ACR.2, FITC-phall and pY assays. Dots represent individual data points, dotted lines represent 95% LA (Limits of Agreement).

## Data Availability

The data and materials are available from the corresponding author on reasonable requests.

## References

[1] Austin CR (1951). Observation on penetration of sperm into the mammalian egg. Aust J Sci Res.

[2] Austin CR (1967). Capacitation of spermatozoa. Int J Fertil.

[3] Chang MC (1951). Fertilizing capacity of spermatozoa deposited into the fallopian tubes. Nature.

[4] Reyes A, Goicoechea B, Rosado A (1978). Calcium ion requirement for rabbit spermatozoal capacitation and enhancement of fertilizing ability by ionophore A23187 and cyclic adenosine 3′:5′-monophosphate. Fertil Steril.

[5] Peknicova J, Moos J, Mollova M, Srsen V, Capkova J (1994). Changes in immunochemical localisation of acrosomal and sperm proteins in boar spermatozoa during capacitation and induced acrosome reaction. Anim Reprod Sci.

[6] Breitbart H, Cohen G, Rubinstein S (2005). Role of actin cytoskeleton in mammalian sperm capacitation and the acrosome reaction. Reproduction..

[7] Dvoráková K, Moore HD, Sebková N, Palecek J (2005). Cytoskeleton localization in the sperm head prior to fertilization. Reproduction..

[8] Pĕknicová J, Kubátová A, Sulimenko V, Dráberová E, Viklický V, Hozák P, Dráber P (2001). Differential subcellular distribution of tubulin epitopes in boar spermatozoa: recognition of class III beta-tubulin epitope in sperm tail. Biol Reprod.

[9] Duncan AE, Fraser LR (1993). Cyclic AMP-dependent phosphorylation of epididymal mouse sperm proteins during capacitation in vitro: identification of an Mr 95 000 phosphotyrosine-containing protein. J Reprod Fertil.

[10] Seshagiri PB, Mariappa D, Aladakatti RH (2007). Tyrosine phosphorylated proteins in mammalian spermatozoa: molecular and functional aspects. J Reprod Fertil Suppl.

[11] Cross NL (1998). Role of cholesterol in sperm capacitation. Biol Reprod.

[12] Ward CR, Storey BT (1984). Determination of the time course of capacitation in mouse spermatozoa using a chlortetracycline fluorescence assay. Dev Biol.

[13] Maxwell WM, Johnson LA (1997). Chlortetracycline analysisof boar spermatozoa after incubation, flow cytometric sorting, cooling, or cryopreservation. Mol Reprod Dev.

[14] Ded L, Dostalova P, Dorosh A, Dvorakova-Hortova K, Peknicova J (2010). Effect of estrogens on boar sperm capacitation in vitro. Reprod Biol Endocrinol.

[15] Brener E, Rubinstein S, Cohen G, Shternall K, Rivlin J, Breitbart H (2003). Remodeling of the actin cytoskeleton during mammalian sperm capacitation and acrosome reaction. Biol Reprod.

[16] Kalab P, Peknicová J, Geussová G, Moos J (1998). Regulation of protein tyrosine phosphorylation in boar sperm through a cAMP-dependent pathway. Mol Reprod Dev.

[17] Piehler E, Petrunkina AM, Ekhlasi-Hundrieser M, Töpfer-Petersen E (2006). Dynamic quantification of the tyrosine phosphorylation of the sperm surface proteins during capacitation. Cytometry A.

[18] Berger T, Turner KO, Meizel S, Hedrick JL (1989). Zona pellucida-induced acrosome reaction in boar sperm. Biol Reprod.

[19] Schmid S, Henning H, Oldenhof H, Wolkers WF, Petrunkina AM, Waberski D (2013). The specific response to capacitating stimuli is a sensitive indicator of chilling injury in hypothermically stored boar spermatozoa. Andrology..

[20] Peknicová J, Moos J (1990). Monoclonal antibodies against boar acrosomal antigens labelling undamaged acrosomes of spermatozoa in immunofluorescence test. Andrologia..

[21] Wang WH, Abeydeera LR, Fraser LR, Niwa K (1995). Functional analysis using chlortetracycline fluorescence and *in vitro* fertilization of frozen-thawed ejaculated boar spermatozoa incubated in a protein-free chemically defined medium. J Reprod Fertil.

[22] Fraser LR, Abeydeera LR, Niwa K (1995). Ca(2+)-regulating mechanisms that modulate bull sperm capacitation and acrosomal exocytosis as determined by chlortetracycline analysis. Mol Reprod Dev.

[23] Martínez-Pastor F, Mata-Campuzano M, Alvarez-Rodríguez M, Alvarez M, Anel L, de Paz P (2010). Probes and techniques for sperm evaluation by flow cytometry. Reprod Domest Anim.

[24] Gil MC, García-Herreros M, Barón FJ, Aparicio IM, Santos AJ, García-Marín LJ (2009). Morphometry of porcine spermatozoa and its functional significance in relation with the motility parameters in fresh semen. Theriogenology..

[25] Maxwell WM, Johnson LA (1997). Chlortetracycline analysis of boar spermatozoa after incubation, flow cytometric sorting, cooling, or cryopreservation. Mol Reprod Dev.

[26] Harkema W, Harrison RA, Miller NG, Topper EK, Woelders H (1998). Enhanced binding of zona pellucida proteins to the acrosomal region of the intact boar spermatozoa in response to fertilizing conditions: a flow cytometric study. Biol Reprod.

[27] Birck A, Labouriau R, Christensen P (2009). Dynamics of the induced acrosome reaction in boar sperm evaluated by flow cytometry. Anim Reprod Sci.

[28] Buckman C, George TC, Friend S, Sutovsky M, Miranda-Vizuete A, Ozanon C, Morrissey P, Sutovsky P (2009). High throughput, parallel imaging and biomarker quantification of human spermatozoa by ImageStream flow cytometry. Syst Biol Reprod Med.

[29] Kerns K, Zigo M, Drobnis EZ, Sutovsky M, Sutovsky P (2018). Zinc ion flux during mammalian sperm capacitation. Nat Commun.

[30] Yeste M, Fernández-Novell JM, Ramió-Lluch L, Estrada E, Rocha LG, Cebrián-Pérez JA, Muiño-Blanco T, Concha II, Ramírez A, Rodríguez-Gil JE (2015). Intracellular calcium movements of boar spermatozoa during ‘in vitro’ capacitation and subsequent acrosome exocytosis follow a multiple-storage place, extracellular calcium-dependent model. Andrology..

[31] Macías-García B, García-Marín LJ, Bragado MJ, González-Fernández L (2019). The calcium-sensing receptor regulates protein tyrosine phosphorylation through PDK1 in boar spermatozoa. Mol Reprod Dev.

[32] Darszon A, Nishigaki T, Wood C, Treviño CL, Felix R, Beltrán C (2005). Calcium channels and Ca2+ fluctuations in sperm physiology. Int Rev Cytol.

[33] Darszon A, Treviño CL, Wood C, Galindo B, Rodríguez-Miranda E, Acevedo JJ, Hernandez-González EO, Beltrán C, Martínez-López P, Nishigaki T (2007). Ion channels in sperm motility and capacitation. Soc Reprod Fertil Suppl.

[34] Calvo L, Dennison-Lagos L, Banks SM, Fugger EF, Sherins RJ (1993). Chemical composition and protein source in the capacitation medium significantly affect the ability of human spermatozoa to undergo follicular fluid induced acrosome reaction. Hum Reprod.

