# Supplementary figures and images for: Fluorescent analysis of boar sperm capacitation process in vitro
Source: Reprod Biol Endocrinol. 2019 Dec 19;17:109. doi: 10.1186/s12958-019-0554-z (PMC6923987; doi:10.1186/s12958-019-0554-z)

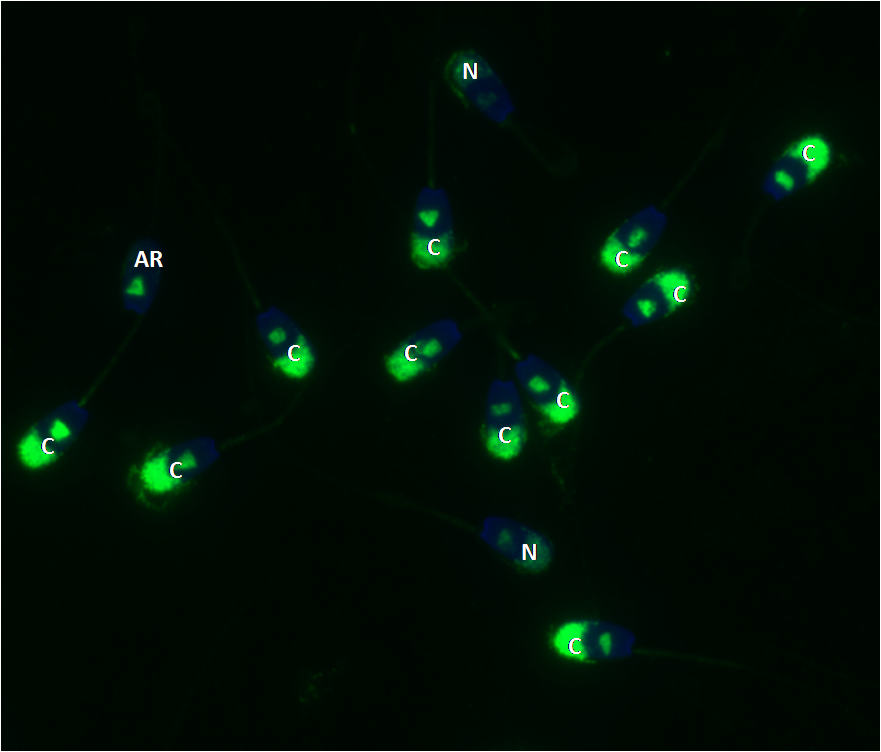

Supplement: Supplementary file 1 — Additional file 1: Figure S1. Example of the fluorescent microscopy analysis of the sperm capacitation status. The microphotograph shows 14 boar sperm stained by anti-pY antibody (green) and counterstained by DAPI. Eleven cells detected as capacitated (C), two cells detected as non-capacitated (NC), one cell as AR. Time 240 min, 400x. [file 12958_2019_554_MOESM1_ESM.tif]

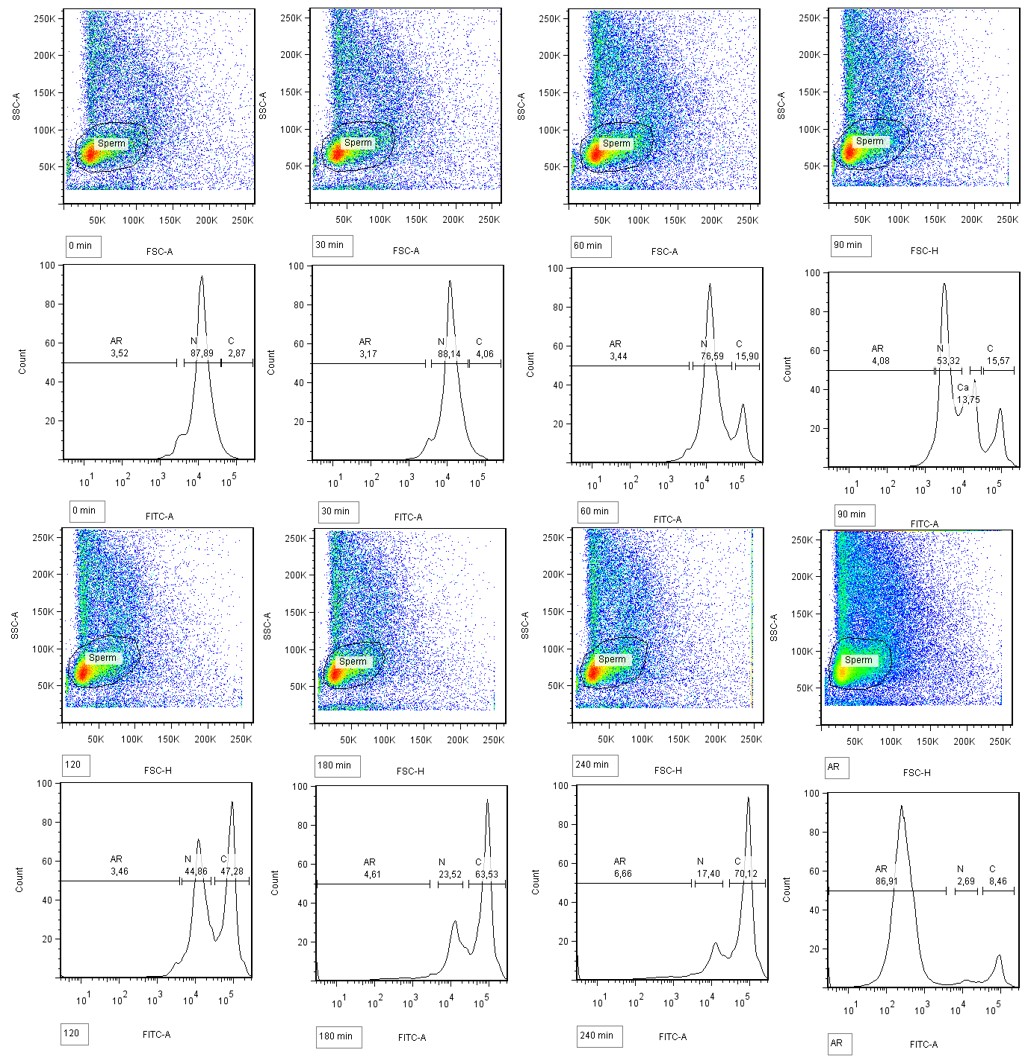

Supplement: Supplementary file 2 — Additional file 2: Figure S2. Example of the flow cytometry analysis of the boar sperm capacitation progress by ACR.2 antibody. The dot plots represent the gating of the analyzed sperm population using FSC and SSC detection and the histograms of the FITC channel with the gating of the non-capacitated (N), capacitated (C) and AR cells (%) for the individual time points (0, 30, 60, 90, 120, 180, 240) and AR. The data from 0, 240 and AR from the 20 individual boars were subsequently used for the preparation of Fig. 3. [file 12958_2019_554_MOESM2_ESM.jpg]

**CTC**

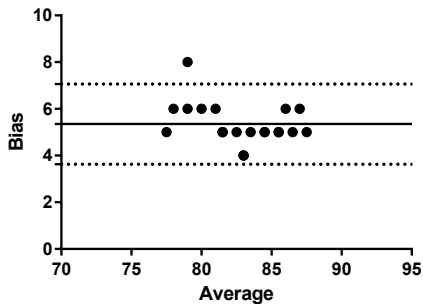

**ACR.2**

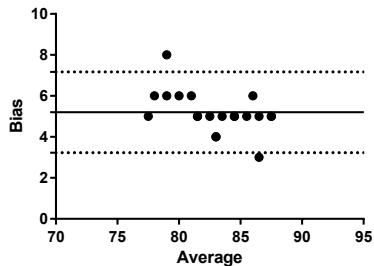

**pY**

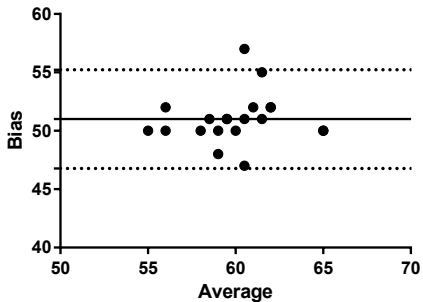

**Phall**

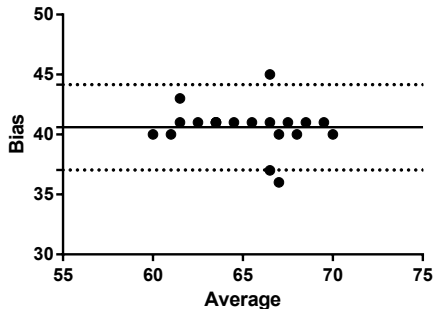

Supplement: Supplementary file 3 — Additional file 3: Figure S3. Bland-Altman plots separately for individual methods. Bland-Altman plots (decomposed Fig. 5) show the absolute bias between the percentage of cells detected as capacitated by individual methods after 240 min of incubation and the percentage of cells detected as acrosome-reacted by PSA after ZP-induced AR. The zero baseline represents the percentage of cells detected as acrosome-reacted by PSA, solid lines shows the absolute biases for CTC, ACR.2, FITC-phall and pY assays. Dots represent individual data points, dotted lines represent 95% LA (Limits of Agreement). [file 12958_2019_554_MOESM3_ESM.pdf]
